# Studies Need More than a Spot Sample: Variability of Urinary Metal Levels over Time

**DOI:** 10.1289/ehp.124-A77

**Published:** 2016-04-01

**Authors:** Julia R. Barrett

**Affiliations:** Julia R. Barrett, MS, ELS, a Madison, WI–based science writer and editor, is a member of the National Association of Science Writers and the Board of Editors in the Life Sciences.

Within the field of environmental epidemiology, researchers often measure biomarkers in urine to estimate how much an individual has been exposed to a particular substance and whether that exposure is associated with a specific health outcome.^1^^,^^2^ However, several studies have indicated that one-time specimens may not provide an accurate basis for characterizing long-term exposures.^1^^,^^3^^,^^4^ A new study in *EHP* reports on the variation of urinary levels of seven metals within a small group of healthy adult Chinese men and demonstrates the value of accounting for variations both among and within individuals to avoid exposure misclassifications.^5^

Urine is the most frequently used specimen type because collection is noninvasive, poses no risk to study participants, and requires little in the way of equipment or expertise. In addition, compared with other specimen types (e.g., blood), relatively large volumes can be collected from a high number of participants; one-time urine samples, known as spot collections, are typically obtained from studies with hundreds or thousands of participants.^1^^,^^2^

**Figure:**
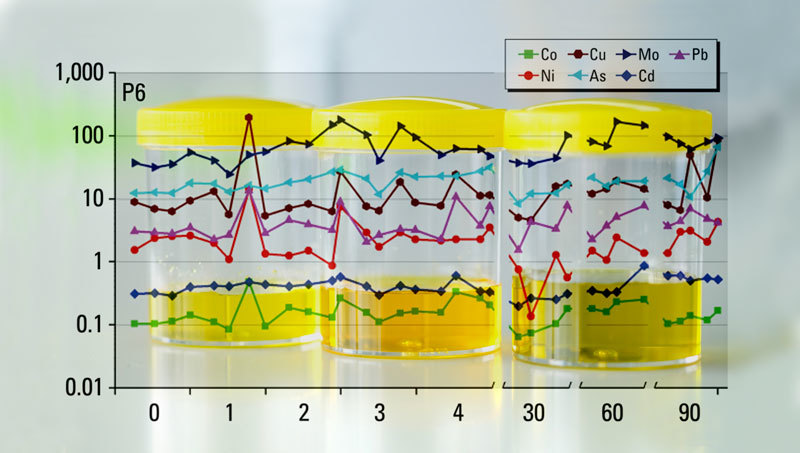
Researchers tracked men’s urinary levels of 7 metals over a 3-month period; this figure shows one participant’s levels over the course of the study. The variation in metal levels over time indicates that single urine samples may not provide an accurate snapshot of an individual’s chronic exposure to metals. Background: © Shutterstock; graph: Wang et al. (2016)^5^

Multiple factors can affect biomarker levels in urine, causing them to vary substantially within the same person from one day to another or even over a single day.^1^^,^^2^^,^^4^ This variation could lead researchers to misclassify overall exposure as either higher or lower than it actually is, which would consequently obscure a relationship between a substance and a health outcome of interest.^3^^,^^5^ “It is critical to consider analyte-specific patterns of variability in urinary metal levels to ensure that epidemiological studies have adequate power to detect exposure–outcome relationships,” says senior author Wen-Qing Lu, a professor of public health at Tongji Medical College in Wuhan, China.

In the current study,^5^ Lu and her colleagues analyzed urine samples for seven metals: arsenic, cadmium, cobalt, copper, lead, molybdenum, and nickel. The samples had come from 11 healthy nonsmoking men in their 20s with no occupational exposure to metals. At eight points within a three-month period (days 0, 1, 2, 3, 4, 30, 60, and 90) the men collected samples each time they urinated in a 24-hour period. For each participant the authors determined metal concentrations of spot samples randomly selected from throughout the day, first-morning samples, and all samples averaged over 24 hours.

The researchers then calculated absolute values and corrected values based on urine concentration (hydration status) and the time since previous void. Values between different people (interindividual) and within each individual (intraindividual) were compared for all samples, for samples collected days apart (on days 0–4), and for samples collected months apart (on days 0, 30, 60, and 90). The authors first classified each participant as having low, medium, or high exposure, based on the average of his spot samples from the entire three-month study period. Then they tested how well values measured in 1–3 randomly selected spot samples predicted whether a man would be classified as having high exposure.

The researchers found detectable levels of all metals in more than 95% of the samples, but only cadmium had a relatively consistent concentration over time. This could be a result of frequent exposure to cadmium, which is found in tobacco and several foods, including rice, potatoes, and leafy vegetables.^6^ Cadmium also has a long half-life of 10–30 years in the body.^7^ For the other six metals, the authors concluded that relying on a single urine sample was likely to lead to exposure misclassification.^5^

“The use of biomarkers to characterize people’s exposure is something that has become a very prominent tool in environmental epidemiology,” says Lesa Aylward, principal at Summit Toxicology LLP. “To have a study like this provide additional information helping us to understand the strengths and limitations of using biomarkers for exposure characterization is really important.” Aylward was not involved with the study.

Because the study was conducted in only a small group of young men, the researchers caution that it may not be generalizable to a broader population. However, Aylward notes that research on variability could be incorporated into current studies. She explains, “It’s not so much that someone needs to go and repeat this in a bigger population, but that people need to recognize the things that are highlighted by this study and turn a critical eye on their own study design.”

## References

[1] Smolders R Inter- and intra-individual variation in urinary biomarker concentrations over a 6-day sampling period. Part 1: Metals. Toxicol Lett 231 2 249 260 2014, doi:10.1016/j.toxlet.2014.08.014 25128590

[2] Aylward LL Sources of variability in biomarker concentrations. J Toxicol Environ Health B 17 1 45 61 2014, doi:10.1080/10937404.2013.864250 24597909

[3] Akerstrom M Variability of urinary cadmium excretion in spot urine samples, first morning voids, and 24 h urine in a healthy non-smoking population: implications for study design. J Expo Sci Environ Epidemiol 24 2 171 179 2014, doi:10.1038/jes.2013.58 24022669

[4] Xiao Q Sources of variability in metabolite measurements from urinary samples. PLoS One 9 5 e95749 2014, doi:10.1371/journal.pone.0095749 24788433 PMC4006796

[5] Wang YX Variability of metal levels in spot, first morning, and 24-hour urine samples over a 3-month period in healthy adult Chinese men. Environ Health Perspect 124 4 468 476 2016, doi:10.1289/ehp.1409551 26372665 PMC4829977

[6] ICdA. Cadmium Exposure and Human Health [website]. Brussels, Belgium:International Cadmium Association (2016). Available: http://www.cadmium.org/environment/cadmium-exposure-and-human-health [accessed 3 March 2016].

[7] Gunier RB Determinants and within-person variability of urine cadmium concentrations among women in Northern California. Environ Health Perspect 121 6 643 649 2013, doi:10.1289/ehp.1205524 23552363 PMC3672909

